# Report of the Convalescent Homes Committee

**Published:** 1918-12-21

**Authors:** 


### REPORT OF THE CONVALESCENT HOMES COMMITTEE

1. The Governors and General Council have this year fixed the sum available for distribution amongst convalescent homes and consumption sanatoria at £10,000, as against £9,000 in 1917. This is an increase of £1,000 on the largest sum ever before placed at the disposal of the Convalescent Homes Committee.

2. The number of applications eligible for consideration amounted to forty-five from convalescent homes and nine from consumption sanatoria, as against forty-one and nine respectively last year.

3. In dealing with all applications, whether from sanatoria or from convalescent homes, the Committee have, as usual, taken into consideration the question of comparative urgency of need, as shown by the audited accounts and balance-sheets of the various institutions to December 31 last. In the case of consumption sanatoria they have also taken note of the proportion of the total work of each institution which is covered by the receipts under the National Insurance Act. As these and other circumstances affecting the relative claims of the different institutions vary from year to year, it must not be assumed that a reduction in the grant to any particular institution, whether for maintenance or for capital purposes, or even the absence of a grant, implies dissatisfaction.

4. The proportion allotted to convalescent homes is again smaller than usual owing to the absence of grants to several homes which since the beginning of the war have ceased to be occupied by convalescent patients from London, and which are therefore temporarily ineligible for consideration by this Committee.

5. The increased amount available for distribution has assisted the Committee to meet the increased demands on the Fund due to the unavoidable rise in the cost of maintenance of beds at sanatoria, and has also enabled them to obtain two additional beds for the use of patients in London hospitals. The accommodation secured this year amounts to sixty-four beds at six sanatoria, situated as follows: Ten beds for children at the Children's Sanatorium, Holt, Norfolk; four beds at the Daneswood Sanatorium (Jewish), Woburn Sands, Beds; ten beds at the Devon and Cornwall Sanatorium, South Brent, Devon; six beds at the Kelling Sanatorium, Holt, Norfolk; twenty-two beds at the Sanatorium of the National Association for the Establishment and Maintenance of Sanatoria for Workers, Benenden, Kent; twelve beds at the Northamptonshire Sanatorium, Creaton, Northants.

6. The Committee recommend that the beds thus reserved be allocated amongst London hospitals as follows: Twelve beds to the City of London Hospital for Diseases of the Chest; two beds to the Royal Hospital for Diseases of the Chest; eleven beds to the London Hospital; ten beds to Guy's Hospital; six beds to the Middlesex Hospital; five beds to St. Thomas's Hospital; four beds to St. George's Hospital; four beds to University College Hospital; two beds to Charing Cross Hospital; two beds to King's College Hospital; two beds to the Royal Free Hospital; two beds to St. Mary's Hospital; two beds to Westminster Hospital.

7. Owing to the continued restrictions on travelling facilities, both by rail and by motor, it has again been found impracticable to arrange for the inspection of consumption sanatoria in the country. All the sanatoria to which grants are recommended had, previous to 1917, been inspected by the Fund's visitors for several successive years.

8. The grants recommended to the various institutions are appended.

For the Committee, WILLIAM H. BENNETT, Chairman.

November 1918.

